# Chloroform

**Published:** 1848-05

**Authors:** 


### CHLOROFORM.

The anæsthetic excitement which prevailed a short time since, has rapidly subsided, as we anticipated it would. The occurrence of fatal consequences in several instances in which ether and chloroform were administered, particularly the latter, has cast a dark shade over the use of these agents. The danger now is, that we shall run into the opposite extreme, and instead of having recourse to these remedies for pain in trifling cases, decline to employ them in those which they may be most necessary and proper.

